# Association between the trajectory of ideal cardiovascular health metrics and incident chronic kidney disease among 27,635 older adults in northern China–a prospective cohort study

**DOI:** 10.1186/s12877-024-04760-5

**Published:** 2024-02-26

**Authors:** Pufei Bai, Xian Shao, Xiaoqun Ning, Xi Jiang, Hongyan Liu, Yao Lin, Fang Hou, Yourui Zhang, Saijun Zhou, Pei Yu

**Affiliations:** 1 NHC Key Laboratory of Hormones and Development, Tianjin Key Laboratory of Metabolic Diseases, Tianjin Institute of Endocrinology, Chu Hsien-I Memorial Hospital, Tianjin Medical University, Tianjin, 300134 China; 2 Special Medical Service Center, Zhujiang Hospital, Southern Medical University, No. 253, Middle Industrial Avenue, Haizhu District, Guangzhou, Guangdong China; 3 Community Health Service Center, Jiefang Road, Tanggu Street, Binhai New District, Tianjin, China

**Keywords:** Cardiovascular health metrics, Chronic kidney disease, Older people, Group-based trajectory model, Prospective cohort study

## Abstract

**Background:**

There is a lack of relevant studies evaluating the long-term impact of cardiovascular health factor (CVH) metrics on chronic kidney disease (CKD).

**Objective:**

This study investigates the long-term change in CVH metrics in older people and explores the relationship between CVH metrics trajectory and CKD.

**Methods:**

In total, 27,635 older people aged over 60 from the community-based Tianjin Chronic Kidney Disease Cohort study were enrolled. The participants completed five annual physical examinations between January 01, 2014, and December 31, 2018, and a subsequent follow-up between January 01, 2019, and December 31, 2021. CVH metrics trajectories were established by the group-based trajectory model to predict CKD risk. The relationships between baseline CVH, CVH change (ΔCVH), and CKD risk were also explored by logistic regression and restricted cubic spline regression model. In addition, likelihood ratio tests were used to compare the goodness of fit of the different models.

**Results:**

Six distinct CVH metrics trajectories were identified among the participants: low-stable (11.19%), low-medium-stable (30.58%), medium-stable (30.54%), medium-high-decreased (5.46%), medium-high-stable (18.93%), and high-stable (3.25%). After adjustment for potential confounders, higher CVH metrics trajectory was associated with decreased risk of CKD (*P* for trend < 0.001). Comparing the high-stable with the low-stable group, the risk of CKD decreased by 46%. All sensitivity analyses, including adjusting for baseline CVH and removing each CVH component from the total CVH, produced consistent results. Furthermore, the likelihood ratio test revealed that the model established by the CVH trajectory fit better than the baseline CVH and Δ CVH.

**Conclusion:**

The higher CVH metrics trajectory and improvement of CVH metrics were associated with decreased risk of CKD. This study emphasized the importance of improving CVH to achieve primary prevention of CKD in older people.

**Supplementary Information:**

The online version contains supplementary material available at 10.1186/s12877-024-04760-5.

## Introduction

The estimated prevalence of chronic kidney disease (CKD) is 13.4% (11.7-15.1%) of the global population [1]. The incidence of CKD increases with age as the estimated glomerular filtration rate (eGFR), used to assess renal function, declines in parallel with age [2]. In the study by Wang et al. [3], the prevalence of CKD in China and the United States was 34.6% and 32.9% (60–89 years), respectively. In Beijing, China, the prevalence of CKD was 20.8% (60–69 years) and 30.5% (≥ 70 years) [4]. The average annual healthcare costs increase with the progress of CKD, and the risk of progression to end-stage renal disease is highest in older people [5]. Therefore, the importance of early prevention and treatment of CKD cannot be overstated.

The American Heart Association (AHA) proposed the ideal cardiovascular health factor (CVH), which is defined as four ideal health behaviors, including no smoking, ideal body mass index (BMI), physical activity, and a healthy diet, and three ideal health factors, including untreated fast blood glucose (FBG) < 100 mg/dL, untreated blood pressure (BP) < 120/80 mmHg, and untreated total cholesterol (TC) < 200 mg/dL [6, 7]. An ideal CVH could reduce the risk of CKD [8, 9]. However, the ability of individuals to reach ideal CVH may vary with aging and lifestyle changes. Besides, most studies have relied on single-time data, whereas the development of CKD takes a long time; hence, assessing their continuous impact is challenging. Currently, few studies have focused on the impact of long-term changes in CVH on the development of CKD risk, especially in the Asian older population. The trajectory model, based on multiple repeated measurements, can assess long-term changes in CVH.

In the present study, we investigated the change patterns of CVH metrics to identify different trajectories of older adults in northern China. In addition, the association between the trajectory of ideal CVH metrics and incident CKD was studied.

## Methods

### Study population

**Fig. 1:**
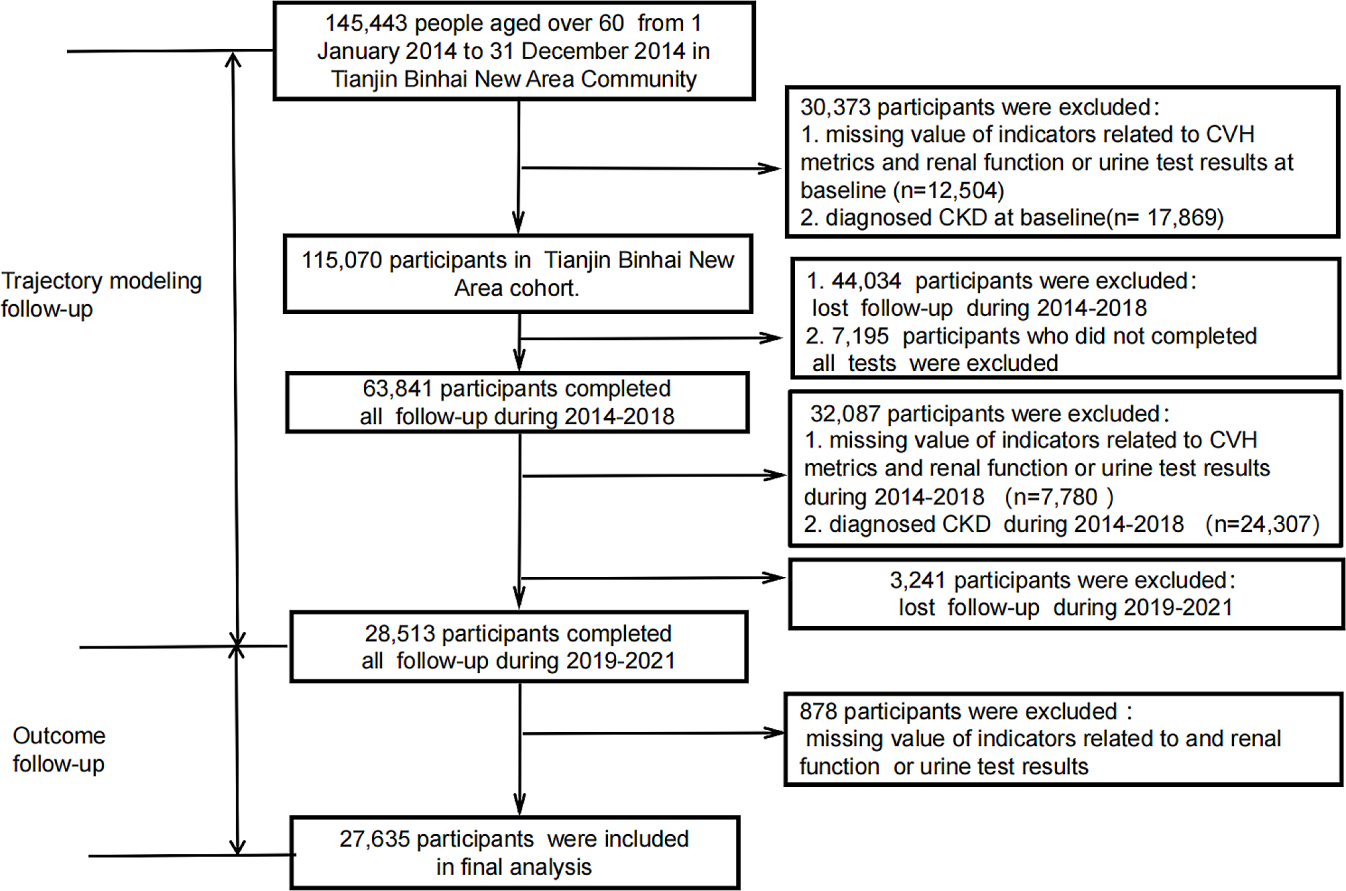
Study flow chart

The Tianjin Chronic Kidney Disease Study is a prospective cohort study in Binhai new area of Tianjin, China. Between January 1, 2014, and December 31, 2014, there were 145,443 older people in the community, most of whom participated in the first visit and completed the questionnaires and health assessments, and subsequently followed up yearly until December 31, 2021. The exclusion criteria were as follows: (1) lost follow-up; (2) missing value of indicators related to CVH metrics and renal function or urine routine results; (3) diagnosed with CKD at baseline; (4) participants reporting incident CKD during CVH metrics trajectory modeling period. After the exclusion of 117,808 participants, 27,635 participants were included in the current analysis (Fig. 1).

### Assessment of CVH metrics

According to the definition of cardiovascular health behaviors and factors proposed by the AHA, the CVH metrics were defined as the sum of seven cardiovascular health behaviors and factor scores (i.e., ideal smoking, ideal BMI, ideal exercise, ideal diet, ideal blood pressure, ideal total cholesterol, and ideal blood glucose) for the evaluation of individual health levels. The BMI was evaluated according to The Guidance for Prevention and Control for Overweight and Obesity from the Ministry of Health. The optimal amount of exercise was defined as 150 min of moderate-intensity exercise weekly. As diet information was not detailed, the consumption of salt and the ratio of meat and vegetable in the diet replaced the food intake categories (details in Supplementary Table 1).

### Assessment of covariates

Physical examinations (i.e., weight, height, BMI, and blood pressure) were conducted by skilled nurses during each survey. The questionnaires were designed to collect demographic characteristics, including age, sex, smoking status, alcohol status, exercise activity (i.e., frequency, way, duration), and medications for diabetes, hypertension, and dyslipidemia. Detection indexes included blood routine (i.e., hemoglobin, platelets, white blood cells, percentage of neutrophil (N%) and lymphocytes (L%)), urine test, blood biochemistry (i.e., triglycerides, total cholesterol, serum creatinine, aspartate aminotransferase, and alanine transaminase), and others (fasting blood glucose).

### Assessment of outcome

The outcome in the present study was defined as the first occurrence of CKD during the follow-up period between 2019 and 2021. Without direct diagnostic data for CKD, we used eGFR as a direct indicator of renal function and proteinuria as a sensitive indicator, according to the Kidney Disease Improving Global Outcomes (KDIGO) 2020 Guidelines [10]. The primary outcome was eGFR < 60 mL/min/1.73 m^2^ and (or) positive urine protein (equal to daily excretion rate of ≥ 150 mg/24 h), and the secondary outcome was rapid decline in renal function (decrease in eGFR more than 5 mL/min/1.73 m^2^ per year). Related participants were suggested to repeat the testing for confirmation and receive advanced medical treatment at advanced healthcare facilities. The eGFR was estimated using the CKD-EPI formula, as previously described [11].

### Statistical analysis

Normally distributed continuous variables were recorded as mean ± SD, and a one-way analysis of variance was used to compare differences between groups. Categorical variables were recorded as numbers (%), and the Chi-square test was used for comparison. Trajectories of CVH metrics were identified with the potential mixture models. The model was evaluated by the Bayesian information standard. The logistics regression model was used to estimate the odds ratios (OR) and 95% confidence interval (CI) of incident CKD according to the CVH trajectories after adjusting for age, gender, HGB, WBC, N%, L%, PLT, TC, TG, ALT, AST, Tbil, and current alcohol use. The sensitivity analysis included: (1) adjusted for baseline CVH metrics; (2) To examine whether this association is largely driven by any of the CVH components, we removed each component from the total CVH metrics. The association between the trajectory of CVH metrics and CKD was then evaluated. The association between the baseline CVH metrics, the annual change in CVH metrics between 2014 and 2018, as secondary exposure, and CKD risk was examined using a logistics regression model. To explore the potential non-linearity of this association, baseline CVH metrics and ΔCVH was used as continuous variables to fit the restricted cubic spline (RCS) model with four knots. The likelihood ratio test was used to compare the fit goodness of the model established by the baseline CVH, Δ CVH, and CVH trajectories. All analyses were completed using R software (version 4.1.0) and STATA (version 17). All statistical tests were two-sided, and *P <* 0.05 was considered significant.

## Results

### Baseline characteristics

**Fig. 2:**
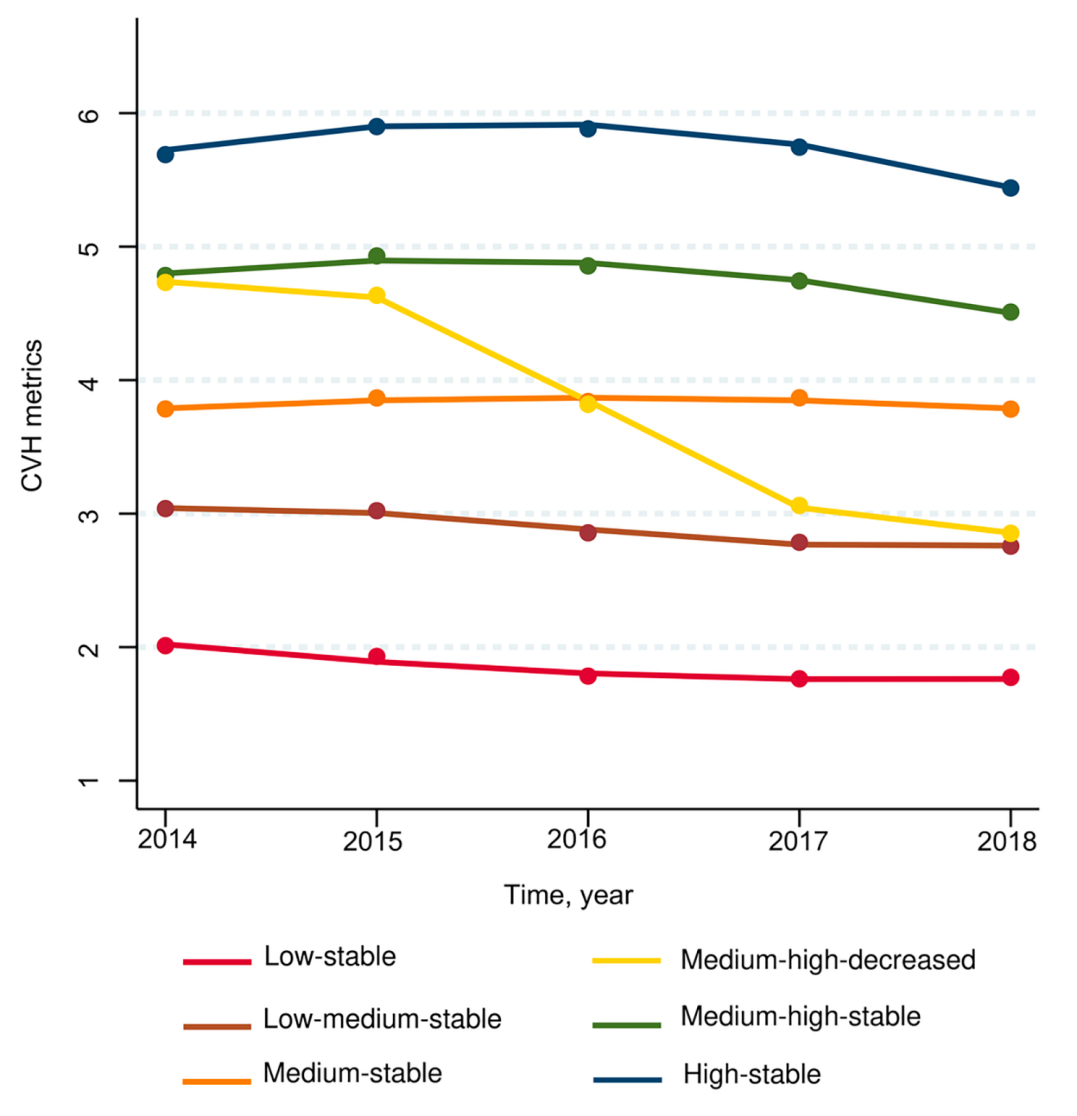
CVH metrics trajectories from 2014 to 2018 established by group-based trajectory model

A total of 27,635 older adults were included in the analyses, with a mean age of 66.1 ± 5.0 years; 47.8% of the participants were male, and 52.2% were female. In total, 3,313 incident CKD events were recorded during the follow-up period of three years. Six distinct trajectory groups were identified with the group base trajectory model: low-stable (*n* = 3093, range: 1.8–2.0), low-medium-stable (*n* = 8456, range: 2.7–3.0), medium-stable (*n* = 8444, range: 3.8–3.9), medium-high-decreased (*n* = 1509, significantly decrease from 4.8 to 2.7), medium-high-stable (*n* = 5235, range: 4.5–5.0), and high-stable (*n* = 898, range: 5.5–6.0). The CVH metrics decreased rapidly during the 2014–2018 period in the medium-high-decreased group while slightly in other trajectory groups (Fig. 2). The demographic characteristics of the participants are presented in Table 1.

**Table 1:** Demographic characteristics of participants

Characteristics	Trajectory group of CVH metrics						*P* value
	Low stable	Low medium	Medium stable	Medium high decreased	Medium high stable	High stable	
No.of participants, n(%)	3093 (11.19%)	8456 (30.58%)	8444 (30.54%)	1509 (5.46%)	5235 (18.93%)	898 (3.25%)	
Men, n (%)	1804 (58.3)	4107 (48.6)	3822 (45.3)	757 (50.2)	2317 (44.3)	391 (43.5)	< 0.001
Age, years	65.7 (4.7)	65.9 (4.8)	66.2 (5.0)	66.2 (5.0)	66.4 (5.3)	66.6 (5.3)	< 0.001
HGB, g/L	142.2 (14.0)	140.3 (13.7)	138.9 (14.0)	139.4 (14.1)	137.4 (13.7)	136.7 (13.7)	< 0.001
WBC × 10^9^/L	6.2 (1.4)	5.9 (1.3)	5.7 (1.3)	5.9 (1.3)	5.5 (1.3)	5.4 (1.3)	< 0.001
N%	55.8 (8.4)	55.6 (8.3)	55.3 (8.4)	55.7 (8.3)	55.2 (8.6)	54.6 (8.8)	< 0.001
L%	35.8 (8.0)	35.6 (8.1)	35.6 (8.1)	35.4 (7.7)	35.6 (8.2)	36.0 (8.5)	0.335
PLT ×10^9^/L	210.2 (52.4)	209.2 (54.5)	206.1 (52.6)	206.4 (52.6)	201.9 (52.3)	201.2 (52.4)	< 0.001
TC, mmol/L	5.6 (0.9)	5.3 (0.9)	5.2 (0.9)	4.9 (0.8)	4.9 (0.9)	4.7 (0.8)	< 0.001
TG, mmol/L	1.9 (1.6)	1.7 (1.3)	1.5 (1.3)	1.5 (1.0)	1.4 (1.3)	1.2 (0.6)	< 0.001
ALT, U/L	21.6 (9.6)	20.6 (9.0)	19.5 (8.7)	20.0 (8.5)	18.6 (8.3)	18.2 (7.7)	< 0.001
AST, U/L	22.5 (8.0)	22.2 (7.4)	21.9 (7.1)	22.2 (6.6)	21.6 (6.9)	21.7 (7.0)	< 0.001
Tbil, umol/L	14.2 (4.6)	14.3 (4.7)	14.4 (4.8)	15.0 (4.6)	14.5 (4.9)	14.5 (5.2)	< 0.001
Alcohol use, n (%)	879 (28.4)	1444 (17.1)	1056 (12.5)	153 (10.1)	386 (7.4)	48 (5.3)	< 0.001
CVH metrics (V1)	2.0 (0.8)	3.0 (0.7)	3.8 (0.7)	4.8 (0.7)	4.8 (0.7)	5.8 (0.8)	< 0.001
CVH metrics (V2)	1.9 (0.7)	3.0 (0.7)	3.8 (0.6)	4.8 (0.7)	5.0 (0.7)	6.0 (0.6)	< 0.001
CVH metrics (V3)	1.8 (0.7)	2.8 (0.6)	3.8 (0.6)	3.8 (0.9)	4.9 (0.6)	6.0 (0.5)	< 0.001
CVH metrics (V4)	1.7 (0.6)	2.8 (0.6)	3.9 (0.6)	2.9 (0.7)	4.8 (0.7)	5.8 (0.6)	< 0.001
CVH metrics (V5)	1.8 (0.7)	2.7 (0.7)	3.8 (0.7)	2.7 (0.7)	4.5 (0.7)	5.5 (0.8)	< 0.001

### Association between the CVH metrics trajectories and risk of CKD

**Fig. 3:**
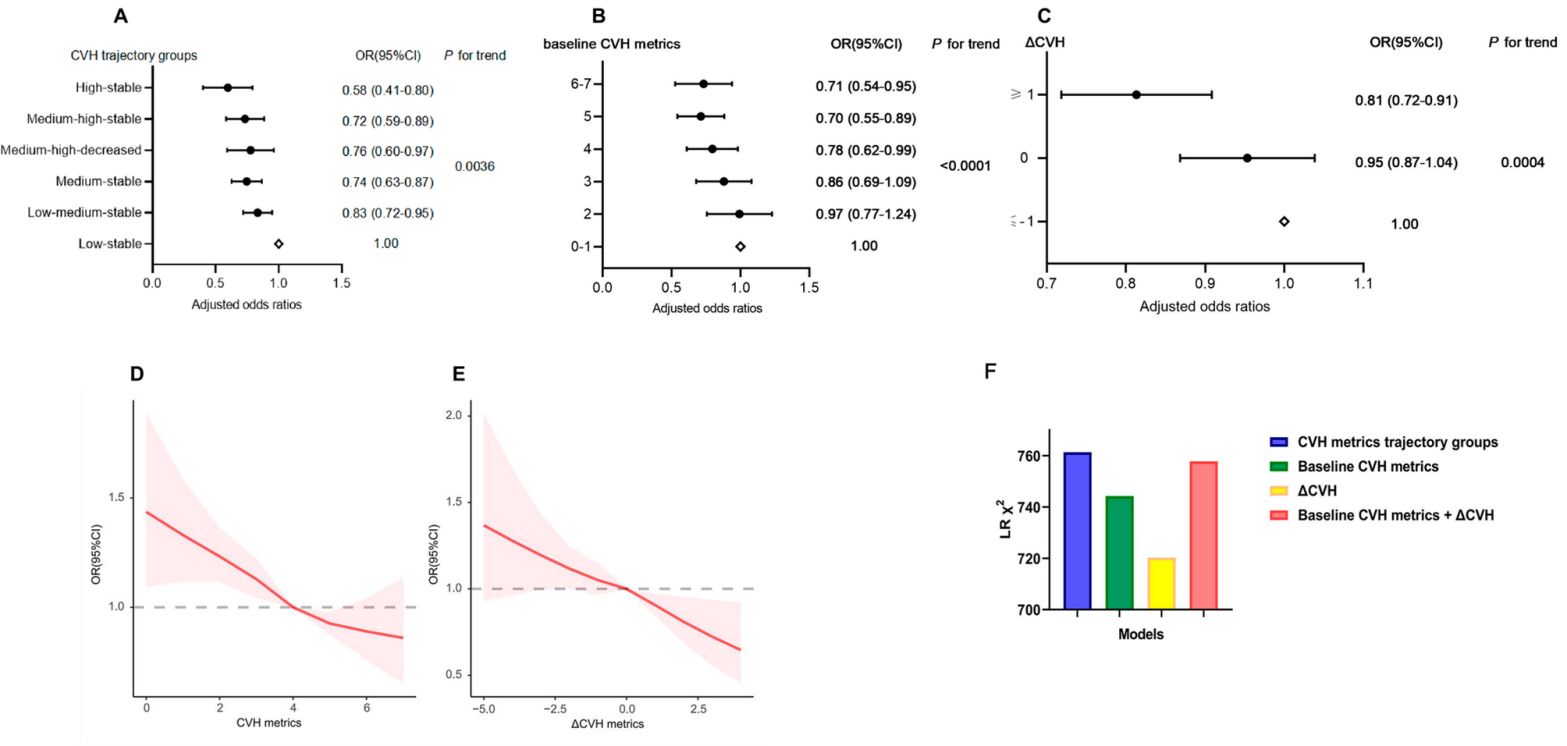
Adjusted odds ratios and 95% confidence intervals of CKD by **(a)** CVH metrics trajectory groups, adjusted for age, sex, HGB, WBC, N%, L%, PLT, TC, TG, ALT, AST, Tbil, current alcohol use, baseline CVH metrics), **(b)** baseline CVH metrics, adjusted forage (y), sex, HGB, WBC, N%, L%, PLT, TC, TG, ALT, AST, Tbil, current alcohol use, and **(c)** Δ CVH metrics, adjusted for age (y), sex, HGB, WBC, N%, L%, PLT, TC, TG, ALT, AST, Tbil, current alcohol use, baseline CVH metrics. **(d)** Restricted cubic spline regression of odds ratios of chronic kidney disease according to the baseline CVH metrics and **(e)** Δ CVH, adjusted for age, gender, HGB, WBC, N%, L%, PLT, TC, TG, ALT, AST, Tbil, current alcohol use (also adjusted the baseline CVH metrics for Δ CVH), shaded area indicates 95% CIs. **(f)** Likelihood ratio test of different regression models,adjusted for age (y), sex, baseline CVH metrics, HGB, WBC, N%, L%, PLT, TC, TG, ALT, AST, Tbil, current alcohol use

The CVH metrics trajectories were significantly associated with the risk of CKD incidence. The risk of CKD decreased as the CVH trajectory improved, ranging from the low-stable to the high-stable groups (Table 2; Figure 3A). After adjusting for potential confounding factors, the ORs (95% CIs) were 0.81 (0.72–0.92) for the low-medium-stable group, 0.71 (0.63–0.81) for the medium-stable group, 0.72 (0.59–0.88) for the medium-high-decreased group, 0.68 (0.59–0.79) for the medium-high-stable group, and 0.54 (0.41–0.70) for the high-stable group, compared with that of the low-stable group (*P* < 0.0001).

**Table 2:** Adjusted odds ratios and 95% confidence intervals of CKD by CVH metrics trajectory groups. Model 1: CVH metrics trajectory groups as the independent variables. Model 2: Included variables in model 1 and adjusted for age(y) and gender. Model 3: Included variables in model 2 and further adjusted for HGB, WBC, N%, L%, PLT, TC, TG, ALT, AST, Tbil, current alcohol use. Model 4: Included variables in model 3 and further adjusted for baseline CVH metrics

CVH trajectory groups	Cases (%)	Model 1	Model 2	Model 3	Model 4
Low-stable	441 (14.3)	1.00	1.00	1.00	1.00
low-medium-stable	1056 (12.5)	0.86 (0.76–0.97)	0.80 (0.71–0.90)	0.81 (0.72–0.92)	0.83 (0.72–0.95)
Medium-stable	962 (11.4)	0.77 (0.69–0.87)	0.69 (0.61–0.78)	0.71 (0.63–0.81)	0.74 (0.63–0.87)
Medium-high-decreased	180 (11.9)	0.81 (0.68–0.98)	0.74 (0.61–0.90)	0.72 (0.59–0.88)	0.76 (0.60–0.97)
Medium-high-stable	587 (11.2)	0.76 (0.67–0.87)	0.66 (0.57–0.75)	0.68 (0.59–0.79)	0.72 (0.59–0.89)
High-stable	87 (9.7)	0.65 (0.50–0.82)	0.54 (0.42–0.69)	0.54 (0.41–0.70)	0.58 (0.41–0.80)
*P* for trend	< 0.0001	< 0.0001	< 0.0001	< 0.0001	0.0036

### Sensibility analysis

To verify the stability of the results, we further explored the association between the CVH trajectory and the second outcome of rapid decline in renal function (eGFR decrease more than 5 mL/min/1.73 m^2^ per year), and consistent results were obtained (Table 3). Besides, to examine whether this association could be explained by a single-time CVH metric, a sensitivity analysis that additionally adjusted for baseline CVH metrics was consistent with the primary analysis (Model 4 in Table 2). We obtained similar results when removing each CVH component from the total CVH metrics and re-established the CVH metrics trajectories (Supplementary Figs. 1–7). In these re-establish models, the CVH metrics trajectories were still associated with risks of CKD incidence after adjusting for potential confounding factors (Table 4).

**Table 3:** Adjusted odds ratios and 95% confidence intervals of rapid decline in renal function by CVH metrics trajectory groups. Model 1: CVH metrics trajectory groups as the independent variables. Model 2: Included variables in model 1 and adjusted for age(y) and gender. Model 3: Included variables in model 2 and further adjusted for HGB, WBC, N%, L%, PLT, TC, TG, ALT, AST, Tbil, current alcohol use. Model 4: Included variables in model 3 and further adjusted for baseline CVH metrics

CVH trajectory groups	Renal function rapid decline events (%)	Model 1	Model 2	Model 3	Model 4
Low-stable	390 (12.6)	1.00	1.00	1.00	1.00
low-medium-stable	1014 (12.0)	0.94(0.83–1.07)	0.92(0.81–1.04)	0.94(0.82–1.06)	0.93(0.81–1.07)
Medium-stable	974 (11.5)	0.90(0.8–1.03)	0.87(0.77–0.99)	0.90(0.79–1.02)	0.89(0.76–1.04)
Medium-high-decreased	620 (11.8)	0.93(0.81–1.07)	0.89(0.77–1.02)	0.93(0.80–1.08)	0.91(0.75–1.12)
Medium-high-stable	153 (10.1)	0.78(0.64–0.95)	0.75(0.61–0.92)	0.76(0.61–0.93)	0.74(0.58–0.96)
High-stable	81 (9.0)	0.69(0.53–0.88)	0.65(0.5–0.84)	0.70(0.53–0.91)	0.69(0.49–0.95)
*P* for trend	< 0.0001	0.0026	0.0003	0.0051	0.0191

**Table 4:** Adjusted odds ratios and 95% confidence intervals of CKD by CVH metrics trajectory groups after removing Individual Cardiovascular health components from the total metrics. Adjusted for age(y), gender, HGB, WBC, N%, L%, PLT, TC, TG, ALT, AST, Tbil, current alcohol use

CVH trajectory groups	Removed component						
	Smoking	Diet	Physical exercise	Blood pressure	Total cholesterol	Fasting blood glucose	BMI
Low-stable	1.00	1.00	1.00	1.00	1.00	1.00	1.00
low-medium-stable	0.95 (0.83–1.09)	0.84 (0.63–1.15)	0.89 (0.71–1.13)	0.87 (0.69–1.10)	0.79 (0.67–0.94)	0.88 (0.75–1.04)	0.90 (0.73–1.11)
Medium-stable	0.80 (0.70–0.92)	0.67 (0.51–0.91)	0.69 (0.55–0.87)	0.65 (0.49–0.87)	0.71 (0.60–0.83)	0.86 (0.73–1.01)	0.79 (0.64–0.98)
Medium-high-decreased	0.78 (0.62–0.97)	0.61 (0.46–0.83)	0.70 (0.52–0.94)	0.75 (0.60–0.94)	0.84 (0.49–1.38)	0.88 (0.70–1.11)	0.79 (0.64–0.98)
Medium-high-stable	0.73 (0.63–0.85)	0.54 (0.40–0.74)	0.65 (0.52–0.82)	0.67 (0.53–0.85)	0.63 (0.53–0.75)	0.79 (0.66–0.95)	0.75 (0.61–0.93)
High-stable	0.63 (0.49–0.79)	0.47 (0.33–0.68)	0.53 (0.41–0.69)	0.61 (0.47–0.79)	0.55 (0.44–0.69)	0.79 (0.62-1.00)	0.65 (0.50–0.85)
*P* for trend	< 0.0001	< 0.0001	< 0.0001	< 0.0001	< 0.0001	0.0076	0.0004

### Association between the baseline CVH metrics and risk of CKD

The baseline CVH metrics were negatively correlated with the risk of CKD. Compared with the 0–1 group, the 2, 3, 4, 5, and 6–7 groups had ORs (95% CIs) of 0.97 (0.77–1.24), 0.86 (0.69–1.09), 0.78 (0.62–0.99), 0.70 (0.55–0.89), and 0.71 (0.54–0.95), respectively (*P* < 0.0001), after adjustment for the confounding factors (Supplementary Tables 2 &Figure 3B). Further, using baseline CVH metrics as a continuous variable, the restricted cubic spline model result displayed that higher baseline CVH metrics were associated with a significantly lower risk of cardiovascular disease (CVD) incidence (Fig. 3D). For one unit of baseline CVH metrics increase, the risk of CKD decreases by 9% (OR 0.91, 0.88–0.94, *P <* 0.0001).

### Association between CVH metrics change in five years and the risk of CKD

The CVH metrics change in five years (ΔCVH) was also negatively correlated with the risk of CKD. Compared with the ΔCVH < 0 group, the ΔCVH = 0 and ΔCVH > 0 groups had ORs (95% CIs) of 0.95 (0.87–1.04) and 0.81 (0.72–0.91), respectively (*P* = 0.0004), after adjusting for the confounding factors (Supplementary Tables 3 &Figure 3C). The restricted cubic spline model demonstrated that increasing ΔCVH were associated with a significantly lower risk of CVD incidence (Fig. 3E). For one unit of ΔCVH increase, the risk of CKD decreases by 7% (OR 0.93, 0.89–0.97, *P* < 0.0001).

### Evaluation of fit performance between different regression models

To compare the goodness of fit between the models established by the CVH metrics trajectory, baseline CVH metrics, and ΔCVH metrics change, the likelihood ratio test depicted that the CVH metrics trajectory and ΔCVH fit better than the baseline CVH metrics (*P* < 0.001) (Supplementary Tables 4 and Fig. 3F).

## Discussion

In this prospective study, six distinct CVH trajectories were identified, which reflected the dynamic changes in cardiovascular health status over time in an older population, which were associated with altered CKD risk. For example, the participants maintaining the highest CVH metrics in the high-stable group had a 46% lower risk of CKD incidence (adjusted OR 0.54, 95% CI: 0.41–0.70) relative to those with a consistently worst health status in the low-stable group, which was independent of the baseline CVH metrics.

It is worth noting that in the medium-high-decreased group, the rapidly decreasing CVH metrics from 4.8 to 2.7 was accompanied by an increasing CKD risk, compared with either the medium-high-stable or medium-stable groups, which offered direct evidence of the deteriorating CVH associated with higher CKD risk.

To the best of our knowledge, this large-scale, community-based study is the first to reveal a potential association between cardiovascular health status change pattern over time and CVD risk in an older population. These observations extend our knowledge of the inverse association between ideal cardiovascular health metrics and a wide range of health outcomes, including myocardial infarction [12], stroke [13], heart failure [14], CVD mortality [15], resistant hypertension [16], diabetes [17], ectopic fat and insulin resistance [18], and venous thromboembolism [19]. The CVH metrics incorporated multiple aspects of health behavior and factors including smoking, diet, exercise, blood pressure, cholesterol levels, glucose and BMI. These indicators were commonly measured and easy to understand. The CVH metrics provided a comprehensive assessment of health and could be applied in large population studies.

The time-cumulative effect of CVH can also impact outcome events [20]. The study by Chung [21] demonstrated that those who maintain higher CVH levels at younger ages would reduce the future risk of CVD incidence or mortality. Similarly, the Korean Genome and Epidemiology Study Ansung-Ansan cohort demonstrated that maintaining good CVH during midlife improves the outcomes for CVD and CKD later [8]. To investigate the time-cumulative effect of CVH, Hou [9] proposed the concept of cumulative CVH score: CVH_1_×time_v1−v2_ + CVH_2_×time_v2−v3_ + CVH_3_×time_v3−v4_. In a fully adjusted model, individuals in the highest quintile had a 75% (95% CI: 66–82%) lower risk of CKD than those in the lowest quintile of cumCVH. Each 1-point-year increase in cumCVH behavioral and factor scores was associated with an 11% (95% CI: 9–13%) reduction in the incidence of CKD.

To date, studies have mostly been based on single-measure data, whereas the progression of chronic disease takes place over a long period. Single-measure data cannot comprehensively evaluate the ongoing impact of CVH metrics on CKD; thus, CVH factors remain poorly understood [22]. For this reason, studies have been initiated to investigate the association between group-based trajectories of CVH and disease risks for individual development. In the Kailuan study [23], five trajectories were classified according to the pattern of changes in cardiovascular health scores (CVH scores) over time among the 74,701 adults included in the study between 2006 and 2010. Compared to the low stability trajectory, the high stability II trajectory was associated with a lower risk of subsequent CVD after adjusting for the correlates (adjusted OR 0.21, 95% CI, 0.16–0.26). Zhang et al. [24] revealed the relationship between the CVH score trajectory and atherosclerosis. Our study further explored the association between CVH trajectory and the risk of CKD in the older population by adopting the group-based trajectory model, which could reflect long-time change patterns and overcome the limitations of single-time measurement data; therefore, it was more objective and reliable in evaluating the impact of CVH on CKD. According to prior epidemiological reports, the incidence of CKD was higher in older people who were also more likely to be in an unhealthy state [25]. CVH metrics, as a comprehensive and convenient tool, could help identify high-risk groups with substandard health behaviors and factors. With the help of CVH metrics being longitudinally and dynamically monitored, the CKD prevalence could be assessed more accurately, which would provide more evidence for strategies of prevention and interventions for CKD, especially in older population.

We also observed a significant inverse dose-response association between the baseline CVH metrics and the change in the CVH metrics with subsequent risk of CKD. On this basis, the likelihood ratio test results depict that the CVH metrics trajectory and CVH metrics change the model fit better than the baseline CVH metrics, which may mean that the CVH trajectory has a unique evaluation value in the comprehensive assessment of individual cardiovascular health status since the CVH trajectory contains information of both baseline level and dynamic changes. Altogether, these data emphasize the importance of public health efforts to improve CVH to prevent the incidence of CKD in older people.

Some strengths of this study are worth mentioning. Our study was a prospective cohort study with a large sample of 27,635 older participants followed for eight years. A trajectory model was established to explore long-term change patterns in CVH metrics. Therefore, we accurately assessed the association between CVH and CKD.

Some limitations also need to be taken into consideration. First, the ideal diet defined by the AHA requires dietary fiber consumption by fruits and vegetables. However, as diet information was not detailed, the consumption of salt and the ratio of meat and vegetable in the diet was used as a replacement. This may cause an underestimation of the dietary factor effect on the risk of CKD. As our study evaluated an older population from Binhai new area, the results need further validation in other populations. Finally, the follow-up period for end-events was relatively shorter, whereas the follow-up period for the CVH metrics was fairly long and we will continue the follow-up in our future work. Besides, we supplemented the secondary outcome of rapid decline in eGFR and obtained consistent results, further increasing the reliability of the results. Although it would be inappropriate to prematurely interpret the association between CVH trajectories and CKD as a causal relationship, the findings of this study provide a new clue for the primary prevention of CKD. CVH metrics, as a comprehensive tool, simple to understand and easy to obtain, could help quickly assess the health status. More importantly, the long-term dynamic monitoring of CVH trajectory showed unique significance, providing more precise evidence on strategies of prevention and interventions of CKD, especially in the older population with a higher prevalence. Although this study was based on a Chinese community, studies from various countries had reported the value of CVH metrics [26–28]. However, research on the CVH metrics trajectory was worth further investigation. Since the indicators were also easy to obtain, research could be applicable and reproducible in other populations and countries. If these results can be replicated in the future, public health will yield more extensive benefits.

## Conclusion

Our study shows that maintaining CVH metrics at a higher level is associated with a lower risk of CKD. In addition, improving CVH metrics can also reduce the risk of CKD. Therefore, regular monitoring of long-term change patterns of cardiovascular health status with the CVH trajectory and improving the overall CVH to achieve the goal of primary prevention of CKD in older people is warranted.

### Electronic supplementary material

Below is the link to the electronic supplementary material.

Supplementary Material 1

## Data Availability

The raw data are not available. However, the data are available from the corresponding author upon reasonable individual request.
