# Supplementary material for: Association between the trajectory of ideal cardiovascular health metrics and incident chronic kidney disease among 27,635 older adults in northern China–a prospective cohort study
Source: BMC Geriatr. 2024 Feb 26;24:193. doi: 10.1186/s12877-024-04760-5 (PMC10898137; doi:10.1186/s12877-024-04760-5)
Supplement: Supplementary file 1 — Supplementary Material 1 [file 12877_2024_4760_MOESM1_ESM.doc]

***Supplementary Material***

**Association between trajectory of ideal cardiovascular health metrics and incident chronic kidney disease among 27635 elderly adults in northern China--a prospective cohort study**

**I Introduction of the Tianjin Chronic Kidney Disease Study**

**II Supplementary Figures**

**Supplementary Figure 1**

**Supplementary Figure 2**

**Supplementary Figure 3**

**Supplementary Figure 4**

**Supplementary Figure 5**

**Supplementary Figure 6**

**Supplementary Figure 7**

**III Supplementary Tables**

**Supplementary Table 1**

**Supplementary Table 2**

**Supplementary Table 3**

**Supplementary Table 4**

**Supplementary Table 5**

**I Introduction of the Tianjin Chronic Kidney Disease Study**

**Cohort name and international clinical trial registration:** Tianjin Chronic Disease Cohort Study; registration number, ChiCTR1900023701.

**Project leader (lead institution):** Pei Yu (Chu Hsien-I Memorial Hospital & Tianjin Institute of Endocrinology, Tianjin Medical University)

**Project description:** The study is an open cohort study that started in January 2013. It aims to explore the intrinsic patterns in the occurrence and progression of chronic diseases in the elderly population, identify key risk factors, and provide a scientific basis for effective prevention and control of the spread of chronic diseases.

**Main objectives:** Prediction, prevention and control strategy development of chronic diseases in the elderly

**Study design:** Open prospective cohort study in adult population.

**Inclusion criteria:** Adults aged 18 years or older.

**Exclusion criteria:** those who were disabled, semi-disabled, refused or unable to participate in regular medical check-up surveys.

**Population characteristics:** Cohort of adults aged 18 years or older from different levels of physical examination centers and communities in Binhai New Area, Tianjin. The population composition was matched for age, gender and occupation.

**Completed participants per year/total number of participants:** more than 300,000/2 million.

**Starting and ending years and follow-up intervals:** 2013 to present; at least 1 follow-up visit every 1 year.

**Medical examination and measurement indicators:** age, gender, smoking, alcohol consumption, occupation, education, blood pressure, heart rate, height, weight, waist circumference, ECG, abdominal ultrasound, blood routine (hemoglobin, platelets, lymphocytes, etc.), urine routine, liver and kidney function, electrolytes, fasting blood glucose, 2h postprandial blood glucose, triglycerides, total cholesterol, HDL, LDL, blood uric acid, albumin, glutamate transaminase, glutamic acid transaminase, etc.; exercise frequency, time and mode, dietary habits, etc.

**Medication indicators:** types of medications for various chronic diseases, medication dosage and frequency, compliance.

**Outcome indicators:** diabetes, hypertension, psychosis, hyperlipidemia, fatty liver, atrial fibrillation, disability and semi-disability, cardiovascular and cerebrovascular diseases, cancer, healthy life expectancy and all-cause mortality, etc.

**Data collection:** Experienced professional physicians and nurses from community and medical examination centers will complete data collection and fill in the system of Tianjin Community Health Service Center.

**Data management:** Uniformly uploaded to system of Tianjin Community Health Service Center for management, and data can be downloaded by logging into this system.

**II Supplementary Figures**


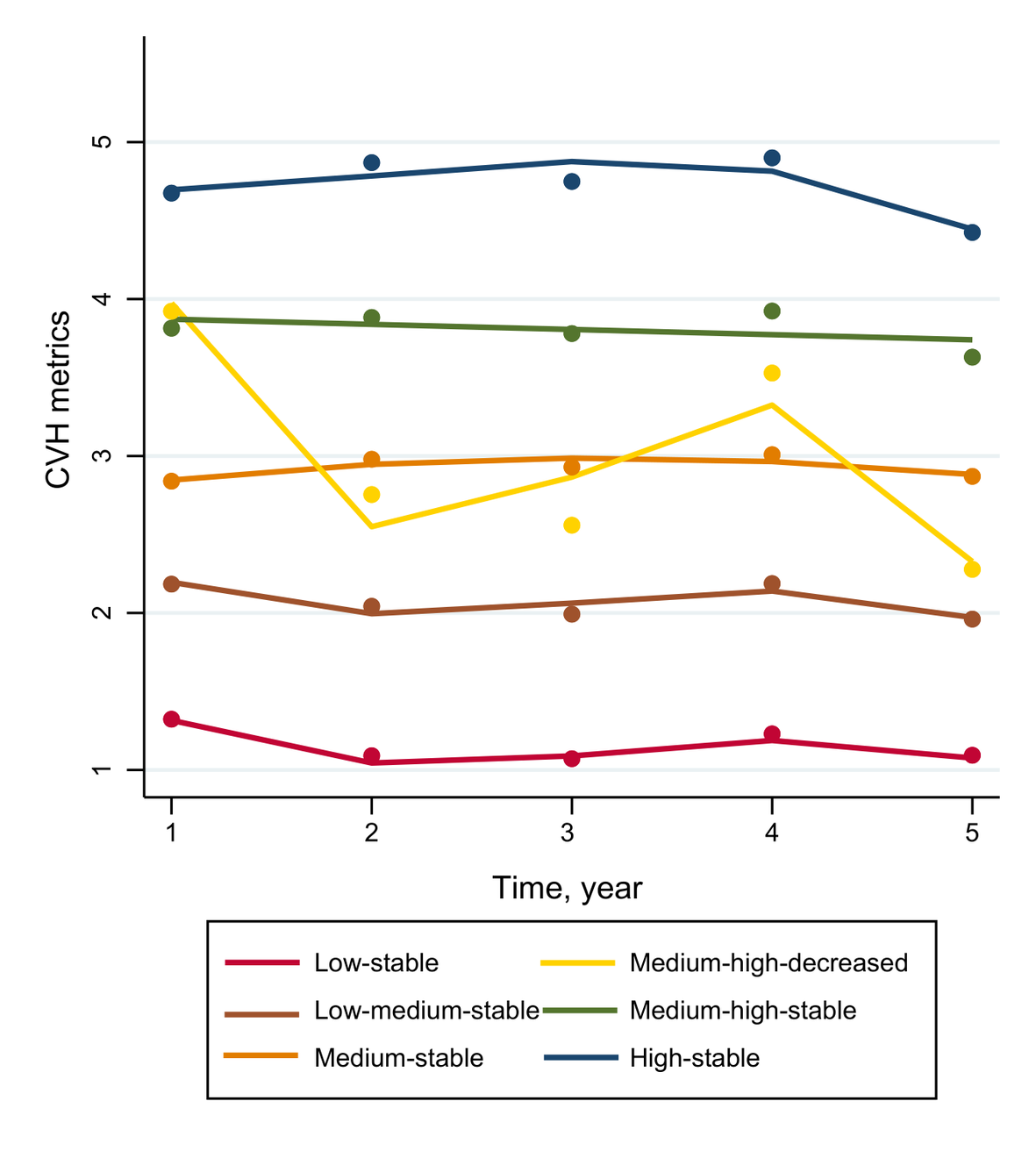


**Supplementary Figure 1. CVH metrics trajectory after removing smoking from the total metrics.**

**
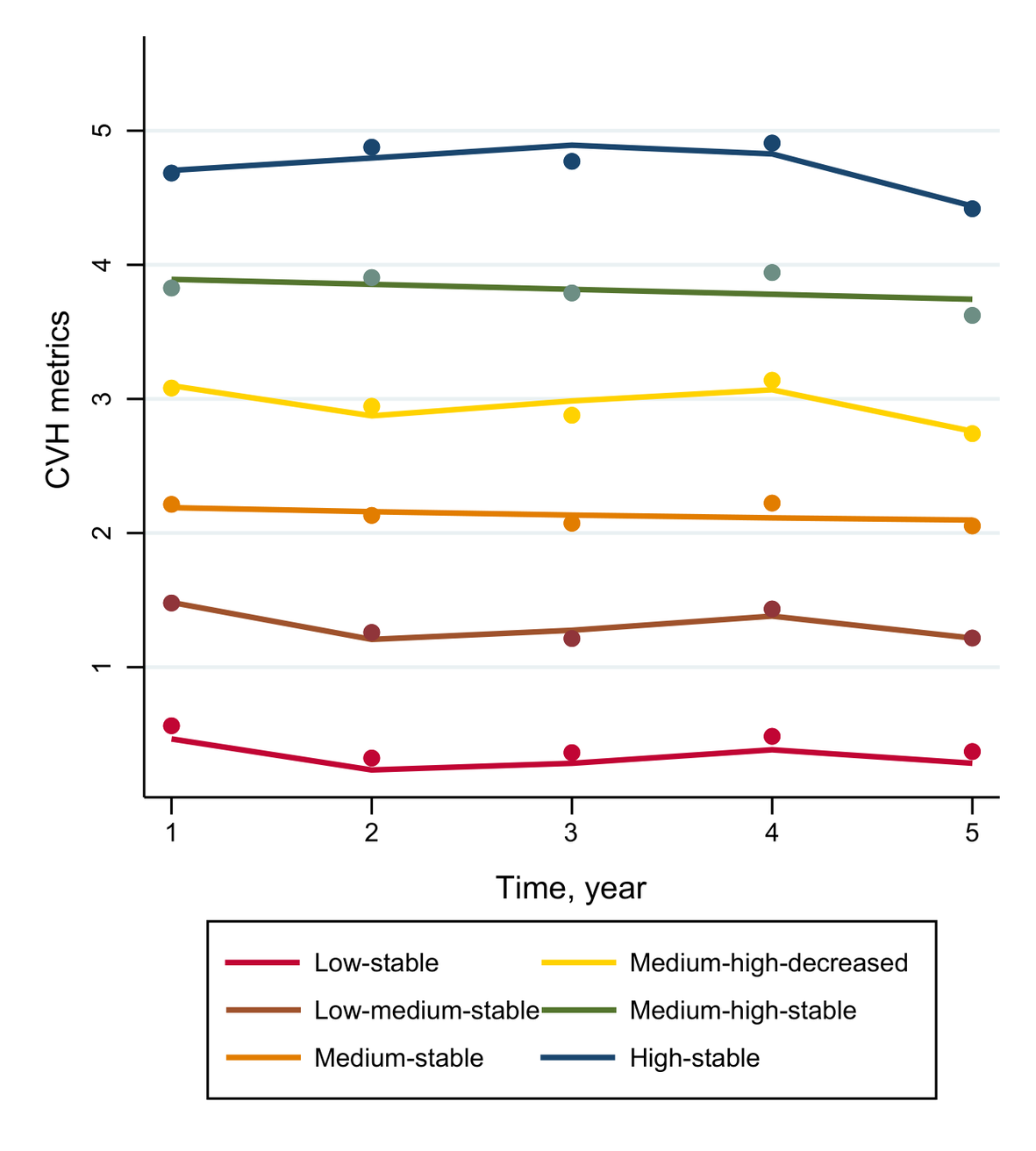
**

**Supplementary Figure 2. CVH metrics trajectory after removing diet from the total metrics.**

**
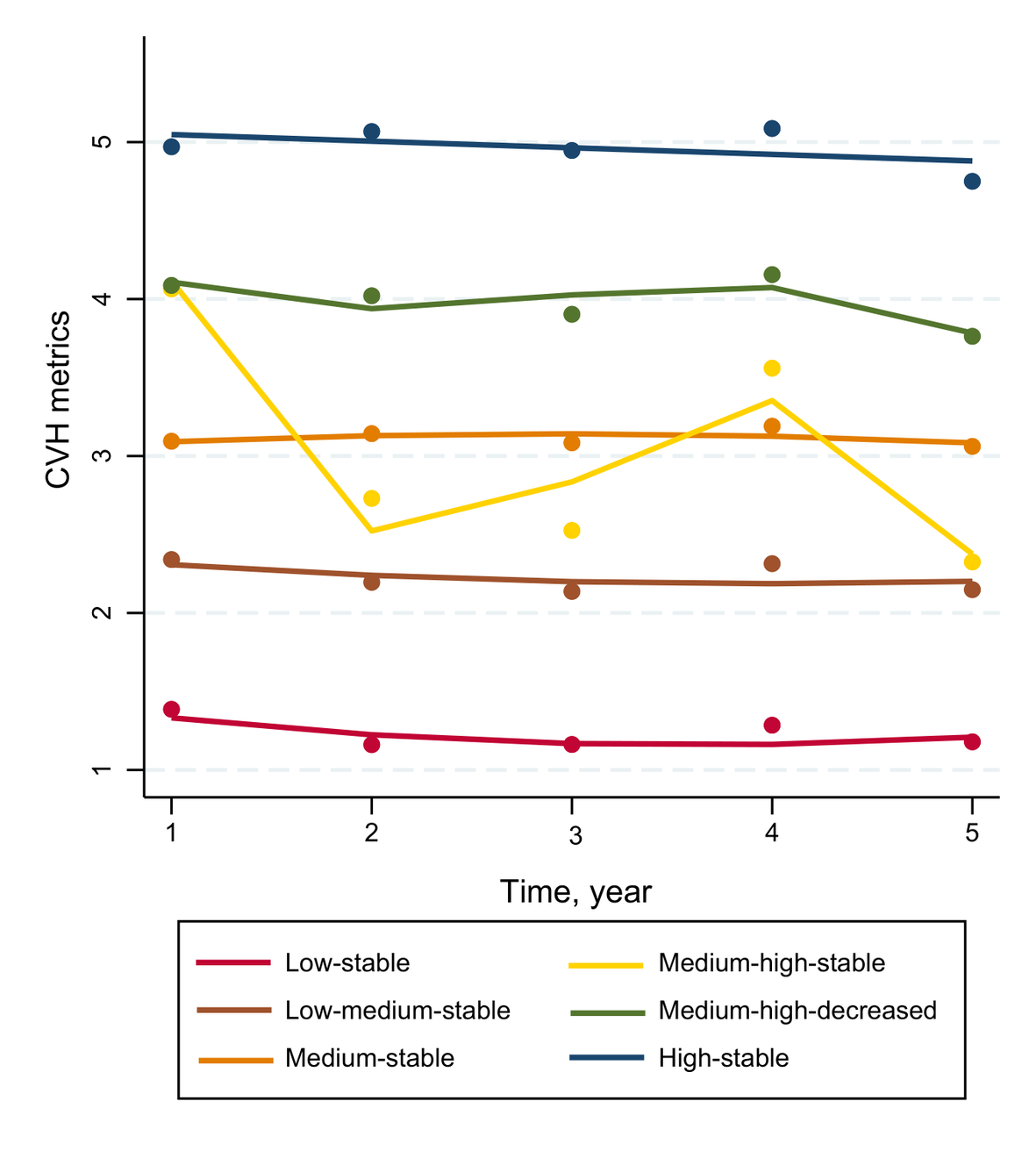
**

**Supplementary Figure 3. CVH metrics trajectory after removing physical exercise from the total metrics.**


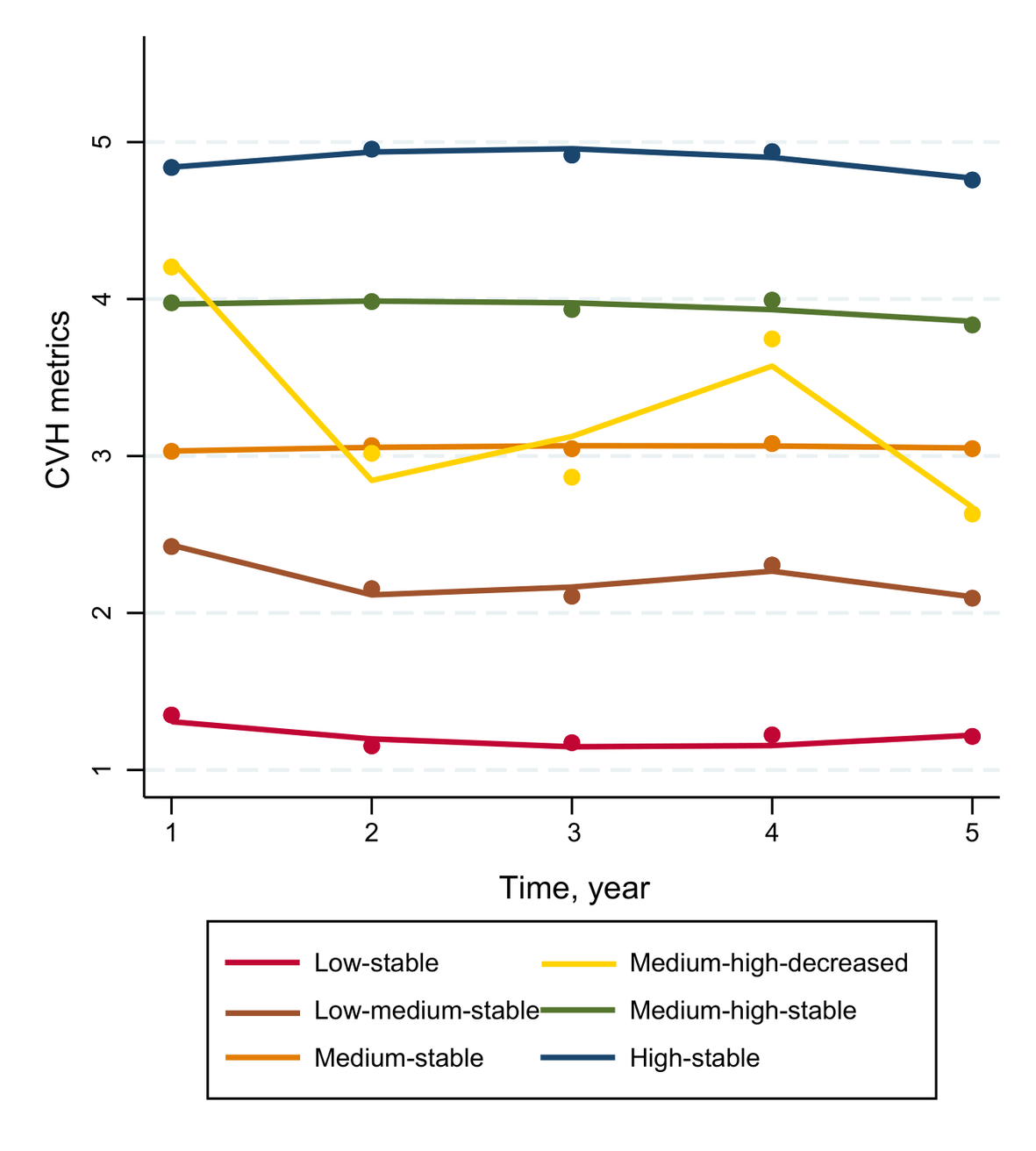


**Supplementary Figure 4. CVH metrics trajectory after removing blood pressure from the total metrics.**


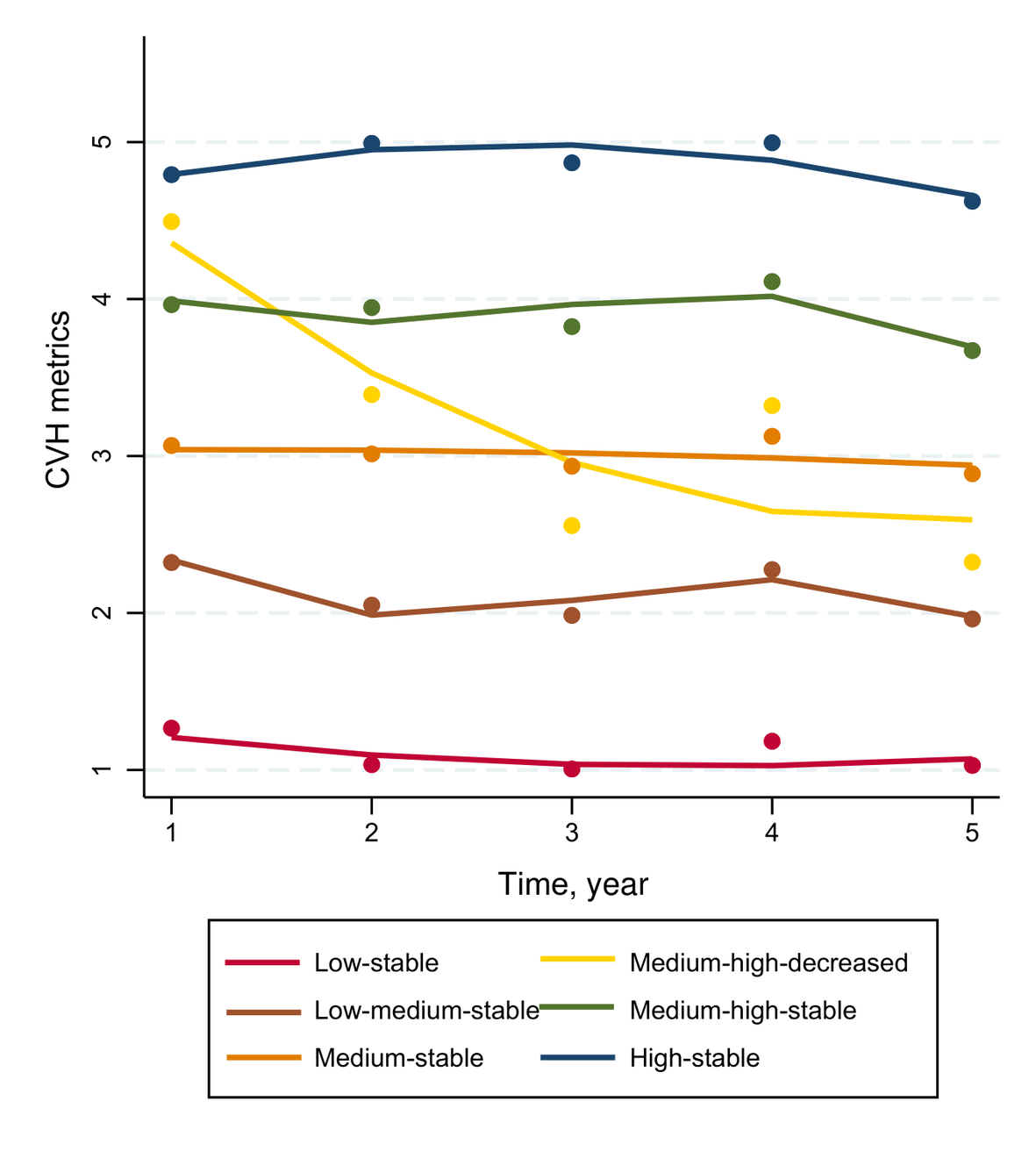


**Supplementary Figure 5. CVH metrics trajectory after removing total cholesterol from the total metrics.**

**
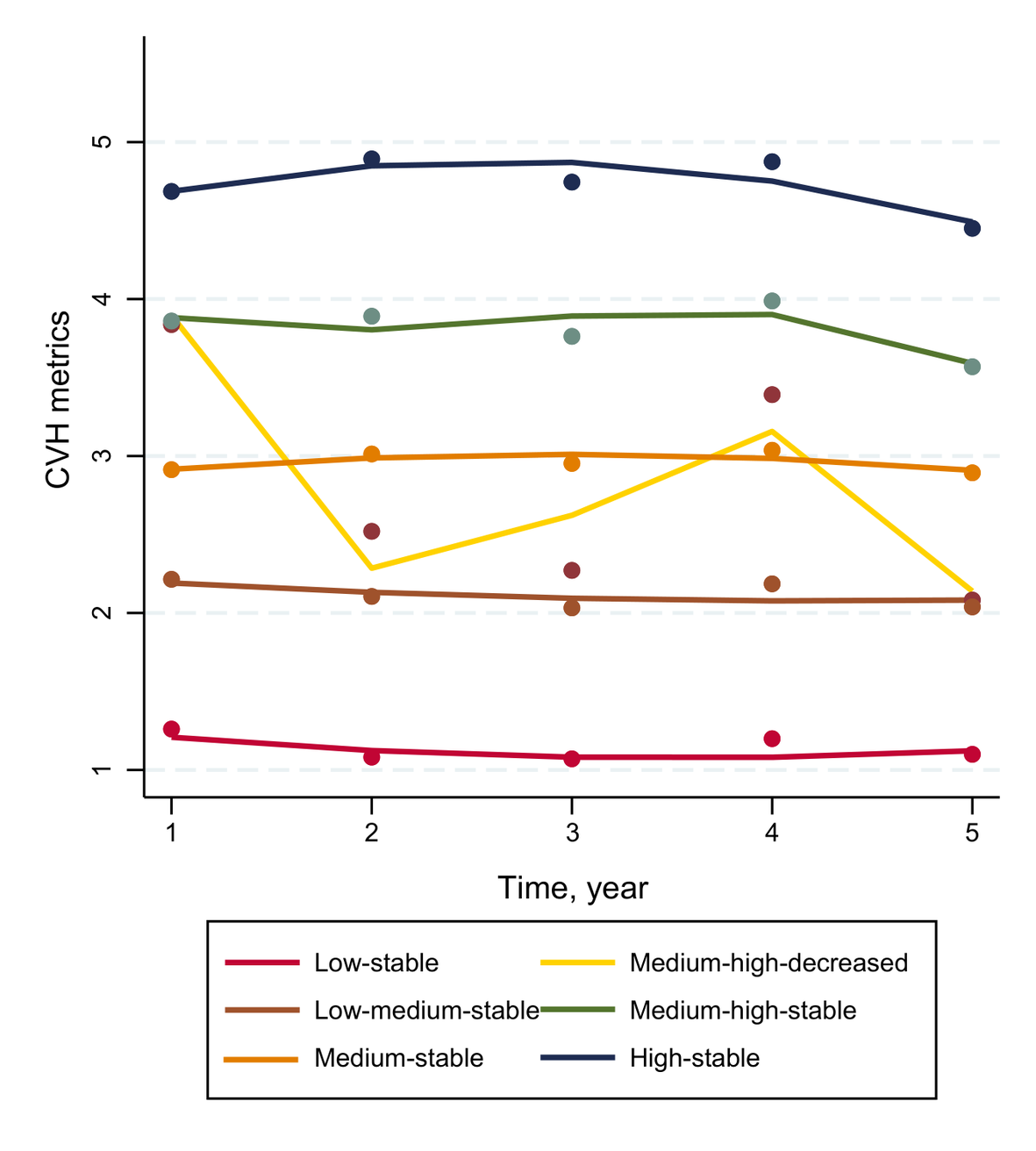
**

**Supplementary Figure 6. CVH metrics trajectory after removing fasting blood glucose from the total metrics.**


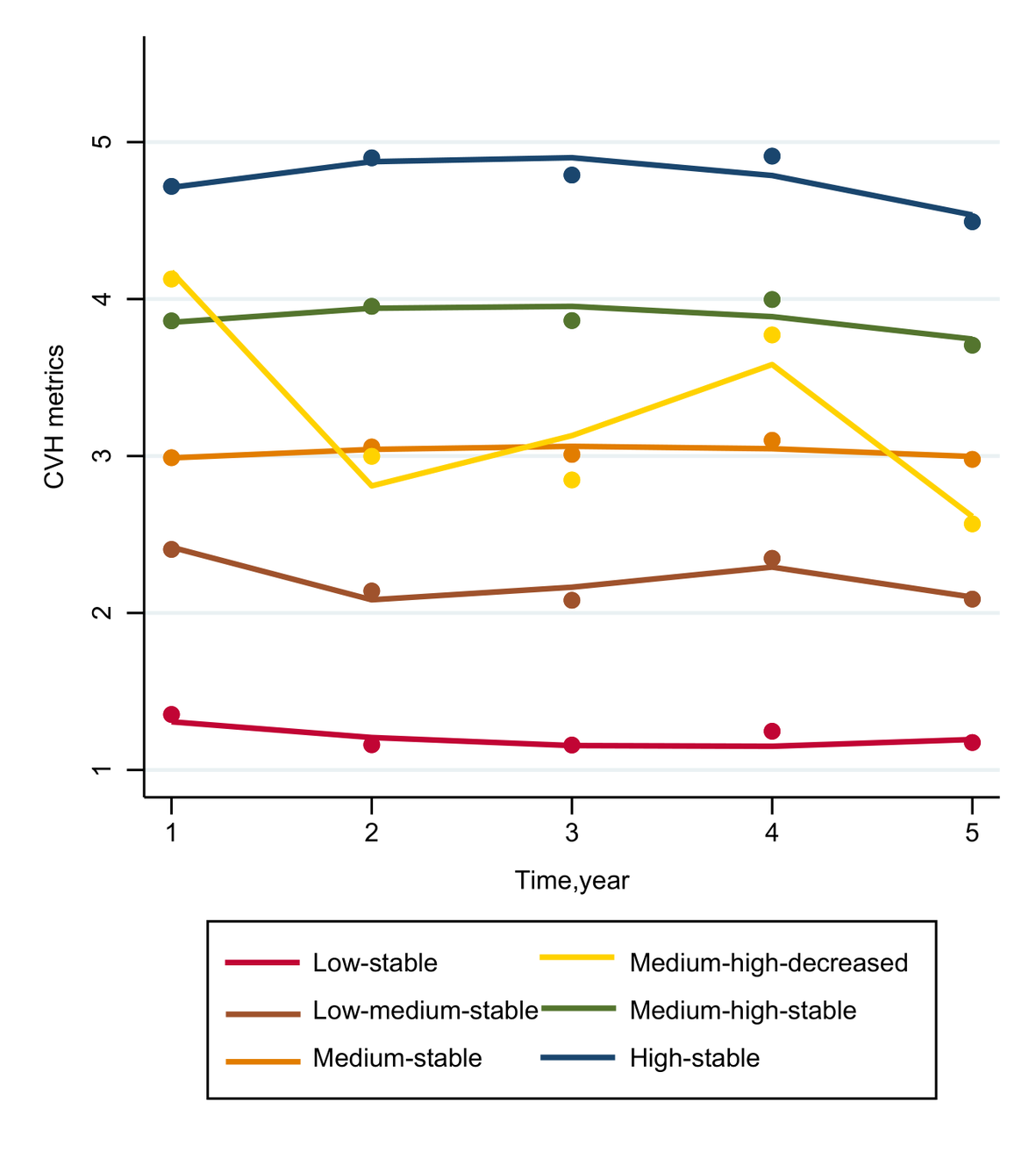


**Supplementary Figure 7. CVH metrics trajectory after removing BMI from the total metrics.**

**III Supplementary Tables**

**Supplementary Table 1. Definitions of ideal cardiovascular health behaviors and factors.**

| Cardiovascular Health Metrics | Intermediate (1) | Poor (0) |
| --- | --- | --- |
| Ideal cardiovascular health behaviors |  |  |
| Smoking | Never | Current or former |
| Diet | A balanced ratio of meat to vegetables and moderate salt intake | Preference for meat or vegetarian or excessive salt |
| Physical exercise (min/w) | ≥150 | <150 |
| Blood pressure (mmHg) | <120/80(untreated) | SBP≥120 or DBP≥80 or treated |
| Total cholesterol (mg/dL) | <200(untreated) | ≥200 or treated |
| Fasting blood glucose (mmol/L) | <5.6(untreated) | ≥5.6 or treated |
| BMI (kg/m^2^) | <24 | ≥24 |

**Supplementary Table 2. Adjusted odds ratios and 95% confidence intervals of CKD by baseline CVH metrics.** Model 1: baseline CVH metrics as the independent variables. Model 2: Included variables in model 1 and adjusted for age (y) and sex. Model 3: Included variables in model 2 and further adjusted for HGB, WBC, N%, L%, PLT, TC, TG, ALT, AST, Tbil, current alcohol use.

| Model | CVH metrics | | | | | | *P* for trend |
| --- | --- | --- | --- | --- | --- | --- | --- |
|  | 0-1 | 2 | 3 | 4 | 5 | 6-7 |  |
| Model 1 | 1.00 | 1.07 (0.85-1.35) | 0.98 (0.79-1.23) | 0.91 (0.74-1.14) | 0.83 (0.66-1.05) | 0.84 (0.65-1.09) | <0.0001 |
| Model 2 | 1.00 | 0.94 (0.74-1.19) | 0.84 (0.67-1.05) | 0.76 (0.61-0.95) | 0.68 (0.54-0.86) | 0.68 (0.53-0.90) | <0.0001 |
| Model 3 | 1.00 | 0.97 (0.77-1.24) | 0.86 (0.69-1.09) | 0.78 (0.62-0.99) | 0.70 (0.55-0.89) | 0.71 (0.54-0.95) | <0.0001 |

**Supplementary Table 3. Adjusted odds ratios and 95% confidence intervals of CKD by Δ CVH metrics. Model 1: baseline CVH metrics plus Δ CVH as the independent variables. Model 2: Included variables in model 1 and adjusted for age (y) and sex. Model 3: Included variables in model 2 and further adjusted for HGB, WBC, N%, L%, PLT, TC, TG, ALT, AST, Tbil, current alcohol use.**

| Model | Δ CVH | | | *P* for trend |
| --- | --- | --- | --- | --- |
|  | ≤ -1 | 0 | ≥ 1 |  |
| Model 1 | 1.00 | 0.97 (0.89-1.05) | 0.83 (0.75-0.93) | 0.0016 |
| Model 2 | 1.00 | 0.92 (0.85-1.01) | 0.78 (0.70-0.87) | <0.0001 |
| Model 3 | 1.00 | 0.95 (0.87-1.04) | 0.81 (0.72-0.91) | 0.0004 |

**Supplementary Table 4. Likelihood ratio test of different regression models.** Model 3：Adjusted for age (y), sex, baseline CVH metrics, HGB, WBC, N%, L%, PLT, TC, TG, ALT, AST, Tbil, current alcohol use.

| Model | LR χ2 | *P* value |
| --- | --- | --- |
| CVH metrics trajectory groups + Model3 | 761.31 | <0.0001 |
| Baseline CVH metrics + Model3 | 744.25 | <0.0001 |
| Δ CVH + Model3 | 720.27 | <0.0001 |
| Baseline CVH metrics + Δ CVH + Model3 | 757.78 | <0.0001 |

**Supplementary Table 5. Univariate logistics regression analysis for risk factors and chronic kidney disease.**

| Characteristics | Estimate | *P* | OR | 95% CI lower | 95% CI upper |
| --- | --- | --- | --- | --- | --- |
| Gender | 0.409759106 | <0.001 | 1.51 | 1.39 | 1.62 |
| Age, years | 0.079231177 | <0.001 | 1.08 | 1.07 | 1.08 |
| HGB, g/L | -0.004281732 | 0.001 | 0.99 | 0.99 | 0.99 |
| WBC × 10^9^/L | 0.074121648 | <0.001 | 1.08 | 1.04 | 1.10 |
| N% | 0.004238578 | 0.058 | 1.00 | 0.99 | 1.00 |
| L% | -0.004372454 | 0.060 | 0.99 | 0.99 | 1.00 |
| PLT, ×10^9^/L | 0.000602713 | 0.083 | 1.00 | 1.00 | 1.00 |
| TC, mmol/L | 0.045375008 | 0.020 | 1.04 | 1.00 | 1.08 |
| TG, mmol/L | 0.039169883 | <0.001 | 1.04 | 1.01 | 1.06 |
| eGFR, mL/min/1.73 m^2^ | -0.025228344 | <0.001 | 0.98 | 0.97 | 0.98 |
| ALT, U/L | 0.001577633 | 0.451 | 1.00 | 0.99 | 1.00 |
| AST, U/L | -0.005024402 | 0.051 | 0.99 | 0.98 | 1.00 |
| Tbil, umol/L | 0.006381823 | 0.102 | 1.01 | 0.99 | 1.01 |
| Alcohol use | -0.369604647 | <0.001 | 0.69 | 0.61 | 0.77 |
| CVH metrics_2014 | -0.064999023 | <0.001 | 0.94 | 0.90 | 0.96 |
| CVH metrics_2015 | -0.076342546 | <0.001 | 0.93 | 0.89 | 0.95 |
| CVH metrics_2016 | -0.058458734 | <0.001 | 0.94 | 0.91 | 0.97 |
| CVH metrics_2017 | -0.075217185 | <0.001 | 0.93 | 0.89 | 0.95 |
| CVH metrics_2018 | -0.076635474 | <0.001 | 0.93 | 0.89 | 0.95 |
|  |  |  |  |  |  |
